# The histone demethylase KDM5C controls female bone mass by promoting energy metabolism in osteoclasts

**DOI:** 10.1126/sciadv.adg0731

**Published:** 2023-04-05

**Authors:** Huadie Liu, Lukai Zhai, Ye Liu, Di Lu, Alexandra Vander Ark, Tao Yang, Connie M. Krawczyk

**Affiliations:** ^1^ Laboratory of Skeletal Biology, Department of Cell Biology, Van Andel Institute, 333 Bostwick Ave NE, Grand Rapids, MI 49503, USA.; ^2^ Department of Metabolism and Nutritional Programming, Van Andel Research Institute, Grand Rapids, MI 49503, USA.

## Abstract

Women experience osteoporosis at higher rates than men. Aside from hormones, the mechanisms driving sex-dependent bone mass regulation are not well understood. Here, we demonstrate that the X-linked H3K4me2/3 demethylase KDM5C regulates sex-specific bone mass. Loss of KDM5C in hematopoietic stem cells or bone marrow monocytes increases bone mass in female but not male mice. Mechanistically, loss of KDM5C impairs the bioenergetic metabolism, resulting in impaired osteoclastogenesis. Treatment with the KDM5 inhibitor reduces osteoclastogenesis and energy metabolism of both female mice and human monocytes. Our report details a sex-dependent mechanism for bone homeostasis, connecting epigenetic regulation to osteoclast metabolism and positions KDM5C as a potential target for future treatment of osteoporosis in women.

## Teaser

KDM5C, an X-linked epigenetic regulator, controls female bone homeostasis by promoting energy metabolism in osteoclasts.

## INTRODUCTION

Bone mass in adults is controlled by the coordination and balance between osteoclast (OC)–mediated bone resorption and osteoblast-mediated bone formation. OCs are multinucleated cells of hematopoietic origin formed through the fusion of mononuclear OC precursors from bone marrow monocytes (BMM). Dysregulation of OC-mediated bone resorption has been associated with bone mass–related diseases, such as osteoporosis. Women have lower average bone mass than men, conferring a two- to fourfold increase in osteoporosis and age-related fractures (*1*). Systemic pathways—in particular, the sex hormones—are prominent in regulating sex-dependent bone mass and have been extensively studied (*2*). However, ex vivo–cultured female OC progenitors are more potent in osteoclastogenesis than male OC progenitors, demonstrating a role for factors intrinsic to OCs regulating sexually dimorphic responses (*3*, *4*). However, the identification of which intrinsic factors that mediate sex-dependent bone homeostasis is still lacking.

Sex chromosomes are the fundamental genetic difference between sexes. Discovering how X- or Y-linked genes contribute to sex-dependent bone mass regulation has the potential to lead to the development of promising therapeutics for osteoporosis in women. An increasing number of studies have revealed that OC differentiation and activity are controlled by epigenetic regulation, largely through controlling the accessibility of transcriptional machinery on key OC genes (*5*, *6*). However, until now, the epigenetic factors that regulate OC differentiation and function have been found to work similarly in both sexes (*5*, *6*). KDM5C (JARID1C/SMCX) is an X-linked lysine H3K4 demethylase that escapes X inactivation, resulting in higher expression in females than males (*7*). Males express a paralog of KDM5C from Chr-Y, KDM5D, which is also a H3K4 demethylase (*8*). Global loss of KDM5C results in spurious transcription of genes normally silenced during development (*9*, *10*). KDM5C has been linked to several sex-dependent phenotypes including X-linked intellectual disability (XLID), autism, X inactivation, adiposity, immune response, and cancer (*11*–*17*). In men and male mice, variants of KDM5C cause XLID with short stature, aggressive behavior, and autism (*18*–*20*). Despite the short stature observed in these individuals, a role for KDM5C in bone homeostasis has not been reported. Here, we report that the loss of KDM5C in BMM increases bone mass in female mice, impairs osteoclastogenesis, and reduces bioenergetic metabolism in OC precursors. Thus, KDM5C represents a cell-intrinsic, sex-dependent epigenetic regulator of osteoclastogenesis and bone mass in females, and its inhibition provides a potential therapeutic strategy for preventing osteoporosis in females.

## RESULTS

### Loss of KDM5C in hematopoietic cells results in increased trabecular bone mass in female mice

**Fig. 1.**
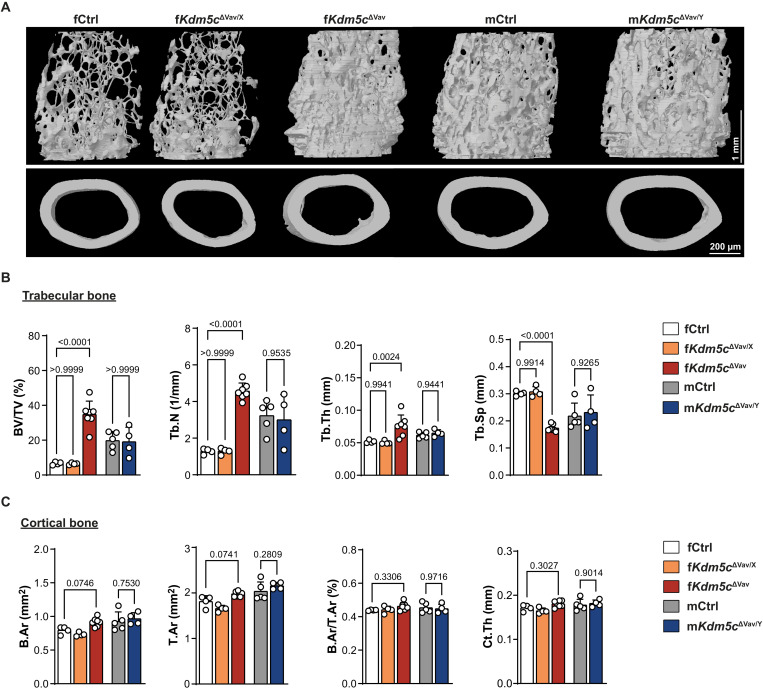
Increased trabecular bone mass in distal femurs of female *Kdm5c*^ΔVav^ but not male *Kdm5c*^ΔVav^ mice. (**A**) Representative micro-CT images and (**B** and **C**) quantitation of femur trabecular bone (top; scale bar, 1 mm) and cortical bone (bottom; scale bar, 200 μm) from 16-week-old mice of indicated genotypes/sex. Data comparisons are conducted using one-way analysis of variance (ANOVA). Each data point represents an individual mouse. Data are presented as means ± SEM.

To investigate the function of KDM5C in the hematopoietic-OC lineage, we generated *Vav-iCre*; *Kdm5c*^fl/fl^ mice, which diminishes KDM5C expression in all hematopoietic cells including BMM that give rise to OCs. No severe global developmental defects were observed in either sex of the KDM5C-conditionally deficient mice. However, upon closer examination, we noticed a marked increase in macroscopic trabecular bone volume in female *Vav-iCre*; *Kdm5c*^fl/fl^ mice (f*Kdm5c*^ΔVav^). When analyzed for bone density and microstructure by microcomputed tomography (micro-CT), bones from f*Kdm5c*^ΔVav^ mice were found to have substantially increased trabecular bone mass at 16 weeks of age (Fig. 1A), as indicated by increased trabecular bone volume/total volume (BV/TV), trabecular number (Tb.N), trabecular thickness (Tb.Th), and decreased trabecular separation (Tb.Sp) (Fig. 1B) compared to female control littermates (fCtrl). In contrast, the male *Vav-iCre*; *Kdm5c*^fl/fl^ mice (m*Kdm5c*^ΔVav/Y^) and female KDM5C heterozygous conditional knockout mice (f*Kdm5c*^ΔVav/X^) have no obvious trabecular bone architecture difference compared to mCtrl and fCtrl, respectively (Fig. 1, A and B). Parameters of cortical bone did not show significant changes between groups (Fig. 1C). These data reveal a sex-dependent role for KDM5C in regulating trabecular bone mass.

### KDM5C intrinsically regulates osteoclastogenesis

**Fig. 2.**
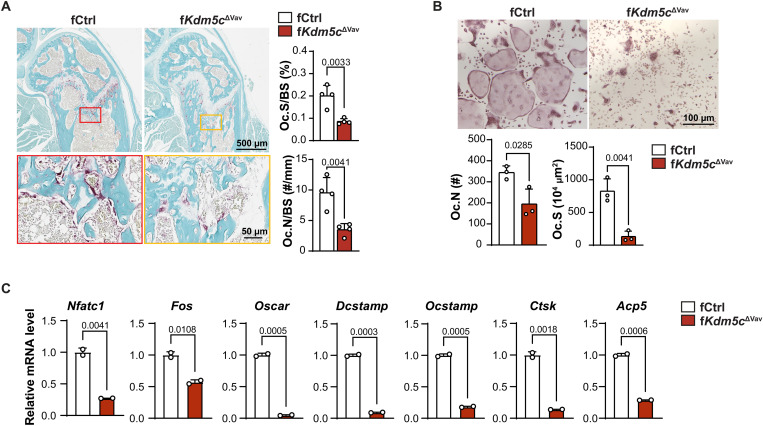
Impaired osteoclastogenesis in female *Kdm5c*^ΔVav^ mice. (**A**) TRAP staining of sectioned femurs from 16-week-old fCtrl and f*Kdm5c*^ΔVav^ mice. Oc.N/BS and Oc.S/BS in trabecular bone area were measured and calculated (*n* = 4 per genotype). Scale bars, 500 μm (top) and 50 μm (bottom). (**B**) Ex vivo osteoclastogenesis on BMM of fCtrl and f*Kdm5c*^ΔVav^ mice. OCs were visualized by TRAP staining 3 to 5 days after differentiation. Oc.N and Oc.S were measured and calculated (*n* = 3 per genotype). Scale bar, 100 μm. (**C**) mRNA levels of OC/osteoclastogenic genes in fCtrl and f*Kdm5c*^ΔVav^ BMM 48 hours after osteoclastogenic induction. mRNA levels were detected by quantitative reverse transcription polymerase chain reaction (qRT-PCR). Relative mRNA levels are shown (*n* = 2 per genotype). All data comparisons are conducted by Student’s *t* test, two-tailed. Data are presented as means ± SEM.

**Fig. 3.**
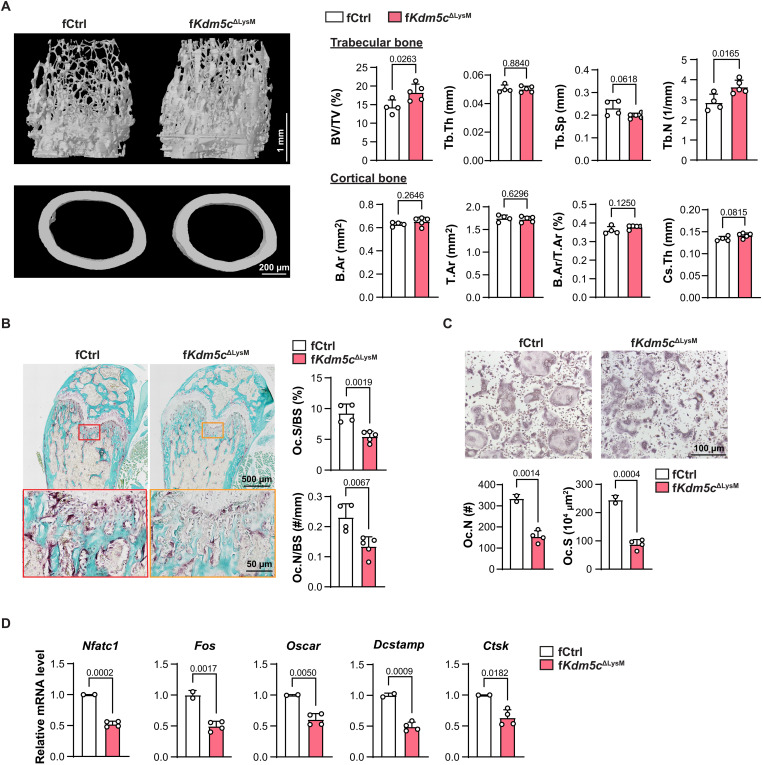
Increased trabecular bone mass and impaired osteoclastogenesis in f*Kdm5c*^ΔLysM^ mice. (**A**) Representative micro-CT images and quantitation of femur trabecular bone (top; scale bar, 1 mm) and cortical bone (bottom; scale bar, 200 μm) from 8-week-old fCtrl and f*Kdm5c*^ΔLysM^ mice. (**B**) Representative TRAP staining images of sectioned femurs from 8-week-old fCtrl and f*Kdm5c*^ΔLysM^ mice, used to calculate Oc.N/BS and Oc.S/BS (*n* = 4 for fCtrl; *n* = 5 for f*Kdm5c*^ΔLysM^). Scale bars, 500 μm (top) and 50 μm (bottom). (**C**) Ex vivo osteoclastogenesis of BMM from fCtrl and f*Kdm5c*^ΔLysM^ mice. OCs were visualized by TRAP staining 3 to 5 days after differentiation. Oc.N and Oc.S were measured and calculated (*n* = 3 per genotype). Scale bar, 100 μm. (**D**) mRNA levels of OC/osteoclastogenic genes were detected by qRT-PCR in fCtrl and f*Kdm5c* ^ΔLysM^ BMM 48 hours after induction. Relative mRNA levels are shown (*n* = 2 for fCtrl, *n* = 4 for f*Kdm5c*^ΔLysM^). All data comparisons are conducted by Student’s *t* test, two-tailed. Data are presented as means ± SEM.

Bone mass in adults is regulated by the coordination and balance between osteoclast-mediated bone resorption and osteoblast-mediated bone formation. We examined bone slices for the presence of OC using tartrate-resistant acid phosphatase (TRAP) staining. We found that the bones from f*Kdm5c*^ΔVav^ mice have significantly decreased OC surface/bone surface (Oc.S/BS) and OC number/bone surface (Oc.N/BS) in trabecular bone compared to controls (Fig. 2A), indicating that osteoclastogenesis and bone resorption are impaired. Other hematopoietic cell types such as T and B cells, which participate in bone mass regulation indirectly by affecting the bone microenvironment (*21*), would also be affected in the *Vav-iCre* mice. To elucidate whether KDM5C regulates bone formation intrinsically in the myeloid lineage, including monocytes and OCs, we isolated BMM from bone marrow of control and f*Kdm5c*^ΔVav^ mice and analyzed their ability to generate OCs ex vivo following RANKL (receptor activator of nuclear factor κB ligand) treatment. OC formation was significantly impaired in f*Kdm5c*^ΔVav^ BMM, as indicated by the reduced Oc.N and Oc.S (Fig. 2B) and decreased expression of osteoclastogenic gene transcripts (Fig. 2C). Furthermore, we generated *LysM-Cre*; *Kdm5c*^fl/fl^ mice, which have *Kdm5c* deleted in myeloid cells. Similar to f*Kdm5c*^ΔVav^ mice, female *LysM*-*Cre*; *Kdm5c*^fl/fl^ mice (f*Kdm5c*^ΔLysM^) had significantly increased trabecular bone mass and decreased OC activity, as indicated by increased BV/TV and Tb.N and decreased Tb.Sp, Oc.S/BS, and Oc.N/BS (Fig. 3, A and B). Consistent with the f*Kdm5c*^ΔVav^ mice, we observed no significant differences in cortical bone mass (Fig. 3A). Overall, the bone phenotypes in the f*Kdm5c*^ΔLysM^ were less severe compared to the f*Kdm5c*^ΔVav^ mice. We found that BMM isolated from f*Kdm5c*^ΔLysM^ mice show significant reduction in osteoclastogenic potential ex vivo, however not to the same extent as observed in the *Kdm5c*^ΔVav^ BMM (Fig. 3, C and D). This is likely due to reduced KDM5C deletion efficiency in f*Kdm5c*^ΔLysM^ BMM compared to f*Kdm5c*^ΔVav^ BMM (fig. S1, A to D). Together, these findings demonstrate that loss of KDM5C intrinsically in myeloid progenitor cells results in reduced osteoclastogenesis.

Next, to investigate whether the intrinsic regulation of KDM5C in osteoclastogenesis is sex dependent, we compared the osteoclastogenic ability between mCtrl (m*Kdm5c*^wt/Y^ *Cre*^+^) and m*Kdm5c*^ΔVav/Y^ BMM. KDM5C-deficient BMM had decreased osteoclastogenesis compared to controls (fig. S2A). The expression of OC genes (*Nfatc1*, *Dcstamp*, and *Acp5*) also decreased in m*Kdm5c*^ΔVav/Y^ cells compared with mCtrl, however to a lesser degree compared to female cells (fig. S2B). This suggests that a compensatory mechanism (e.g., KDM5D) is present in male BMM for osteoclastogenesis. Thus, KDM5C controls bone mass in a sex-specific manner due to its differential ability to control osteoclastogenesis in male versus female BMM.

### Mitochondrial respiration and energy metabolism are impaired following the loss of KDM5C in BMM

**Fig. 4.**
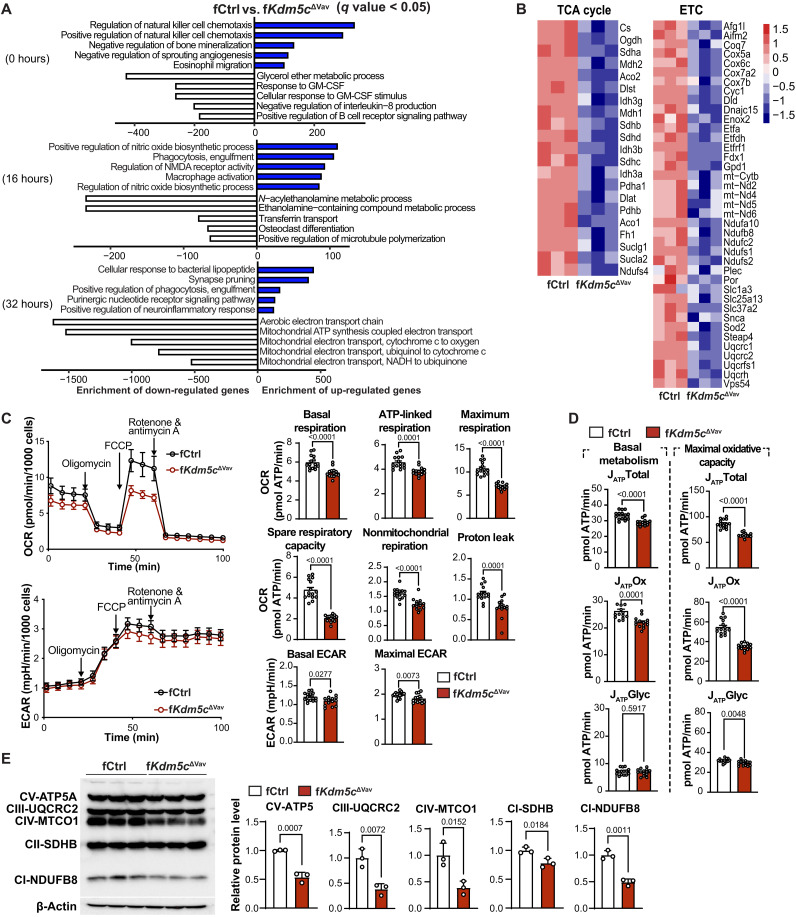
KDM5C-deficient BMM have decreased bioenergetic metabolism during osteoclastogenesis. (**A**) The top five enriched gene ontology biological process terms assigned to up-regulated (blue bars) or down-regulated (white bars) genes in f*Kdm5c*^ΔVav^ versus fCtrl cells during osteoclastogenesis. Gene expression was determined by RNA-seq at three time points during osteoclastogenesis. Genes with false discovery rate < 0.05 were chosen for analysis. (**B**) Heatmap of TCA cycle genes and mitochondrial electron transport chain (ETC) genes expression in f*Kdm5c*^ΔVav^ and control BMM after osteoclastogenic induction. (**C**) OCR (top) and ECAR (bottom) in fCtrl and f*Kdm5c*^ΔVav^ BMM. OCR and ECAR were detected 72 hours after osteoclastogenic induction. Detailed parameters including basal and maximum respiration, ATP-coupled respiration, spare respiratory capacity, nonmitochondrial respiration, and proton leak are shown in the bottom. (**D**) Oxidative ATP production rate and maximum glycolytic ATP production in f*Kdm5c*^ΔVav^ and control BMM. (**E**) Immunoblot of mitochondrial OXPHOS complex proteins (left) and densitometry quantifications (right) of control and *Kdm5c*-deficient BMM stimulated by RANKL for 72 hours. Each lane represents one mouse (*n* = 3 per genotype). All data comparisons are conducted by Student’s *t* test, two-tailed. Data are presented as means ± SEM.

KDM5C not only represses transcription via demethylation of H3K4me2/3 at gene promoters but also promotes gene expression in mouse embryonic stem cells (*10*, *15*) and ERα-positive breast cancer cells (*22*) by converting H3K4me2/3 modifications into H3K4me1 or recruiting transcription factors on specific transcriptional enhancers. Therefore, to investigate how KDM5C affects transcriptional programming of BMM, we compared gene expression of fCtrl and f*Kdm5c*^ΔVav^ BMM at different stages of osteoclastogenesis ex vivo (0, 16, and 32 hours). Significant transcriptomic differences were observed between fCtrl and f*Kdm5c*^ΔVav^ at all three stages (fig. S3). Using Gene Ontology analysis, we found that genes that were increased in KDM5C-deficient BMM/OCs were related to immune cell activity and inflammation, while genes with decreased expression were enriched in metabolic and mitochondrial respiration–related pathways (Fig. 4A). Notably, mitochondrial adenosine triphosphate (ATP) synthesis and electron transport pathways are repressed the most in KDM5C-deficient cells at early stages of OC formation (Fig. 4B, 32 hours). These data demonstrate that KDM5C promotes the expression of genes related to mitochondrial metabolism in BMM/OC.

Osteoclastogenesis and osteolysis are energy-consuming processes (*23*), and preosteoclast differentiation and OC survival are suppressed when mitochondrial metabolism is impaired (*23*–*25*). We found that mitochondrial metabolism genes increased significantly over the course of OC differentiation, supporting the essential role of mitochondrial metabolism in osteoclastogenesis (fig. S4, A and B). Therefore, to test whether decreased gene expression alters mitochondrial bioenergetic metabolism, we measured oxygen consumption rate (OCR) and extracellular acidification rate (ECAR) as surrogates of mitochondrial respiration and glucose fermentation (conversion of pyruvate to lactate), respectively (Fig. 4C). Notably, basal respiration, ATP-coupled respiration, spare respiratory capacity, and maximal respiratory capacity were all significantly reduced in KDM5C-deficient BMM compared to controls (Fig. 4C). We also found that KDM5C-deficient BMM have reduced glucose fermentation (Fig. 4C). These results demonstrate that KDM5C positively regulates bioenergetic metabolism. This was confirmed by the decrease in ATP production from oxidative phosphorylation (OXPHOS) and glycolysis (Fig. 4D). Our results also confirm previous results that OCs generate most of their ATP via OXPHOS (Fig. 4D) (*24*). We examined the expression of proteins involved in electron transport chain (ETC) assembly and function and found that ATP5, UQCRC2, MTCO1, SDHB, and NDUFB8 were all reduced in the KDM5C-deficient cells relative to controls (Fig. 4E and fig. S5A). Consist with *Vav-iCr*e genes involved in ETC assembly and function including *Atp6vod2*, *Uqcrc2*, *Atp1b3*, *Cox6b1*, *Sdhb*, and *Sdha* had lower expression in *LysM-Cre* KDM5C–deficient BMM (fig. S5B). Similar to OC genes (fig. S2B), the expression of mitochondrial genes was decreased in male KDM5C–deficient cells but to a lesser extent than observed in females (fig.S5D). To examine whether specific complexes of the ETC were differentially essential for osteoclastogenesis, we inhibited complex I, II, or III, using piericidin, atpenin A5, or myxothiazol/antimycin, respectively. We found that inhibition of any of the complexes completely blocked OC formation with no TRAP-positive cells compared to control group [dimethyl sulfoxide (DMSO) treatment] (fig. S6). These results indicate that mitochondrial ETC is essential for osteoclastogenesis. Overall, these data demonstrate that KDM5C is necessary for bioenergetic metabolism in female BMM/OC, largely through promoting mitochondrial respiration.

### KDM5C-regulated osteoclastogenesis is mediated in part by PGC-1β

**Fig. 5.**
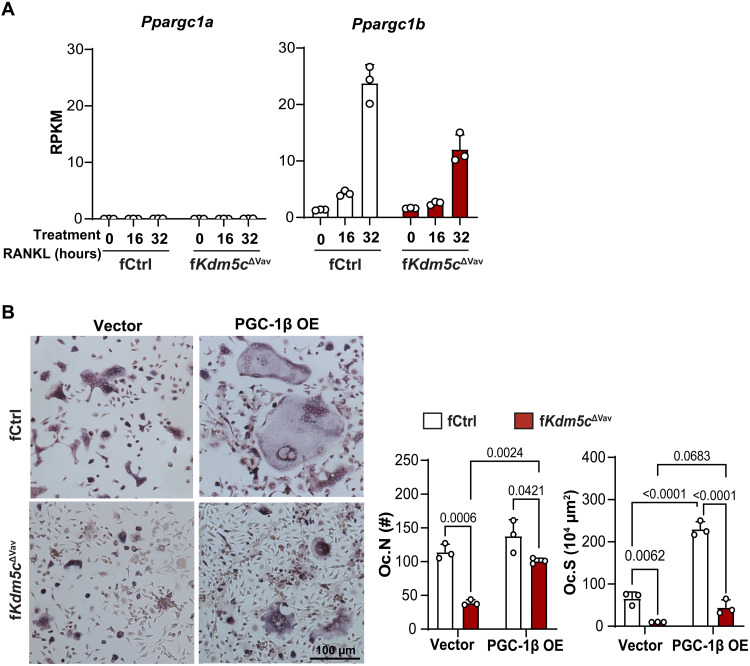
Partial rescue of osteoclastogenesis by PGC-1β. (**A**) *Ppargc1b* and *Ppargc1a* expression (RNA-seq) during ex vivo osteoclastogenesis assay at indicated time points. (**B**) Ex vivo osteoclastogenesis measured by TRAP staining of BMM from fCtrl and f*Kdm5c*^ΔVav^ mice transduced with control or PGC-1β overexpression vector (two-way ANOVA analyses). Scale bar, 100 μm. Data are presented as means ± SEM.

Next, we investigated how KDM5C loss alters metabolic programming in the BMM/OC population. PGC-1α and PGC-1β are transcriptional coactivators that promote mitochondrial biogenesis and the expression of mitochondrial metabolism genes (*26*). Previous studies reported that PGC-1β supports OC formation (*27*, *28*) by enhancing mitochondrial biogenesis and cytoskeletal rearrangement (*29*). We found that PGC-1β (*Ppargc1b*), but not PGC1-α (*Ppargc1a*), was highly expressed in control BMM and increased during osteoclastogenesis (Fig. 5A). We found that PGC-1β expression did not increase to the same extent in KDM5C-deficient BMM/OC (Fig. 5A and fig. S5C). To determine whether increasing the expression of PGC-1β in KDM5C-deficient BMM could rescue osteoclastogenesis, we overexpressed PGC-1β in control and KDM5C-deficient BMM using retroviral expression. We found that the osteoclastogenic potential of KDM5C-deficient BMM could be partially restored by PGC-1β, as indicated by increased Oc.N and Oc.S in an ex vivo osteoclastogenesis assay (Fig. 5B). PGC-1β–mediated regulation of mitochondrial metabolism has been shown to be dependent on iron uptake by the transferrin receptor protein 1 (TfR1) (*27*), and iron itself is required for synthesis of cofactors essential to the function of enzymes in the ETC. We examined the expression of *Tfrc* (the gene encoding TfR1) in KDM5C-deficient BMM/OC and found decreased expression relative to controls (fig. S7). These data show that PGC-1β mediates KDM5C-regulated osteoclastogenesis, but other factors, including iron uptake via TfR1 and/or the expression of other mitochondrial genes, are also likely contributing.

### KDM5 inhibition dampens osteoclastogenic potential of both mouse and human monocytes

**Fig. 6.**
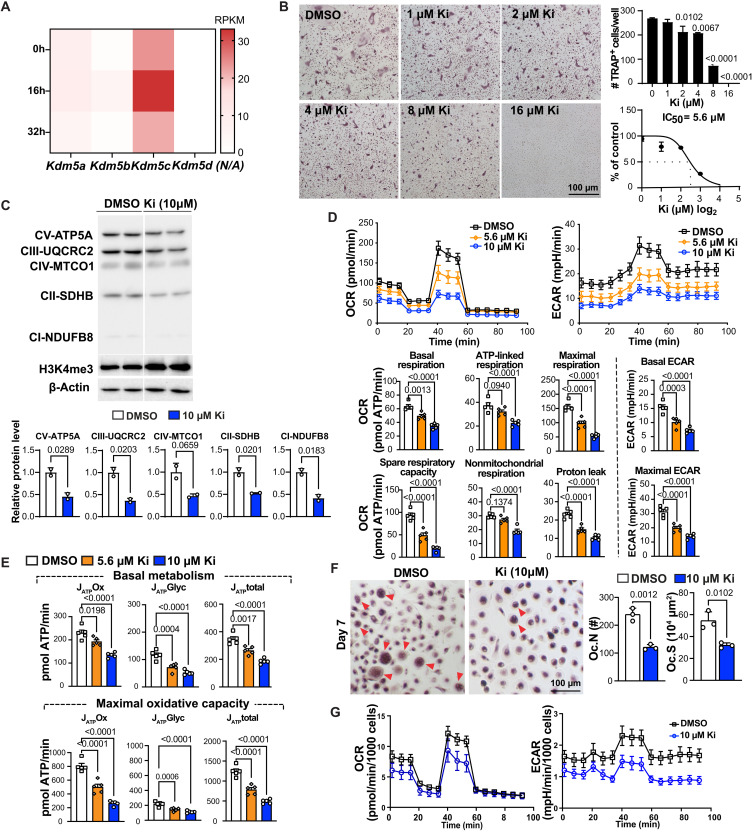
Pharmacological inhibition of KDM5 impairs mitochondrial metabolism and osteoclastogenesis of mice and human monocytes. (**A**) The gene expression of KDM5 family members during osteoclastogenesis (analyzed by RNA-seq, as in Fig. 5A). (**B**) Representative TRAP staining images of BMM osteoclastogenesis following treatments with different doses of KDM5 inhibitor (Ki) (top). The number of TRAP-positive cells per well was quantified and used to calculate IC_50_ of KDM5i in osteoclastogenesis (bottom). Scale bar, 100 μm. (**C**) Western blots of mitochondrial OXPHOS proteins and H3K4me3 after Ki (10 μM) treatment. (**D**) OCR (left) and ECAR (right) of RANKL-stimulated (72 hours) BMM treated with DMSO, 5.6 or 10 μM Ki. All parameters were calculated as in Fig. 4. (**E**) Oxidative and glycolytic ATP production of BMM following Ki treatment. (**F**) Human PBMC osteoclastogenesis with Ki treatment. Monocytes cultured from human PBMCs were treated with RANKL for 7 days before TRAP staining. Multinuclear (>3) OCs are indicated by red arrowheads. Total numbers (Oc.N) and total area (Oc.S) of OCs were quantified using ImageJ (*n* = 3 for each group). Scale bar, 100 μm. (**G**) OCR (left) and ECAR (right) of human PBMC monocytes treated with DMSO and 10 μM Ki 5 days after osteoclastogenic induction. Comparisons in (C) and (F) are conducted by Student’s *t* test, two-tailed; in (D) and (E), by one-way ANOVA analyses. Data are presented as means ± SEM.

Next, we tested whether we could prevent osteoclastogenesis in fCtrl BMM by using small-molecule inhibitors of KDM5C demethylase activity. While no KDM5C-specific inhibitor exists, pan-KDM5 inhibitors (KDM5i) are available and have been used in clinical trials for the treatment of cancer and hepatitis B (*30*, *31*). Our RNA sequencing (RNA-seq) data show that *Kdm5c* is expressed more highly than other KDM5 family members in female BMM (Fig. 6A), suggesting that KDM5C may have a greater role than other KDM5 members in regulating osteoclastogenesis. We found that KDM5i dose-dependently suppressed RANKL-induced osteoclastogenesis of female BMM, indicated by TRAP staining in an ex vivo osteoclastogenesis assay, with a median inhibitory concentration (IC_50_) of 5.6 μM (Fig. 6B). Consistently, KDM5i treatment significantly down-regulated key mitochondria OXPHOS complex proteins (Fig. 6C) and mRNAs (*Sdhb*, *Atp6v0d2*, *Sdha, Uqcrc2*, and *Uqcrfs1*), as well as mRNA expression of OC marker genes (*Dcstamp*, *Acp5*, and *Ctsk*) and *Ppargc1b* (fig. S8A). OCR, ECAR, and ATP production were also impaired by KDM5i in a dose-dependent manner (Fig. 6, D and E), demonstrating that the function of KDM5C in regulating mitochondrial metabolism is dependent on its demethylase activity. KDM5i increased H3K4me3 level in CD14^+^ monocytes of human peripheral blood mononuclear cells (PBMCs) (fig. S8B). The effect of KDM5i on osteoclastogenesis and energy metabolism is conserved in female mice and humans, indicated by the reduction in the number and area of multinuclear OCs (Fig. 6F) and the impairment in OCR, ECAR, and ATP production (Fig. 6G and fig. S8C) in KDM5i-treated female peripheral human blood monocytes under osteoclastogenic differentiation. Thus, targeting KDM5C has the potential to reduce OC function therapeutically, potentially mitigating bone loss in females.

## DISCUSSION

Our work has identified KDM5C, an X-linked chromatin-modifying enzyme, as a female-specific regulator of bone mass that promotes OC differentiation and function. Females, without KDM5C in BMM/OC, displayed increased bone mass comparable to control or KDM5C-deficient males demonstrating that KDM5C uniquely regulates bone mass in females. Mechanistically, we found that KDM5C promotes bioenergetic metabolism required for osteoclastogenesis. Thus, we have identified a mechanism linking epigenetic and metabolic programming of OCs to sex-specific bone mass regulation. Females are at a higher risk than males for developing osteoporosis, mainly because of the inherent lower peak bone mass before menopause and a more aggressive bone loss at postmenopause stage. Estrogen not only inhibits OC formation and function and was used for osteoporosis treatment for women but also leads to strong side effects, including cancers. Current treatment alternatives to estrogen have been developed and successfully used to treat osteoporosis; however, none of these therapeutics target a mechanism specific to females. Our study showed that pharmacological inhibition of KDM5C blocks osteoclastogenesis of both human and mouse monocytes, indicating that KDM5C is a viable therapeutic target for osteoporosis, particularly for females.

While KDM5C predominantly functions as a transcriptional repressor by removing active H3K4me2/3 marks from promoters, it can also stimulate gene expression dependent on or independent of demethylase activity (*10*, *15*, *22*). Our data show that KDM5C promotes mitochondrial metabolism and related gene expression. In *Drosophila*, the KDM5A-C ortholog KDM5 (Lid) also promotes the transcription of genes important for mitochondrial function (*32*). KDM5 regulation of mitochondrial gene expression is mediated by the PHD3 domain, a domain that is present in KDM5A/B but not KDM5C/D (*32*). In our study, use of the KDM5-specific histone lysine demethylase inhibitor reduces mitochondrial metabolism and related gene expression, demonstrating that KDM5C demethylase activity is critical for BMM/OC bioenergetic metabolism. Demethylase-dependent positive regulation of gene expression by KDM5C occurs by trimming H3k4me2/3 for optimal enhancer activity (*10*). Thus, KDM5C may control the mitochondrial transcriptional program in OCs through enhancer regulation. In particular, *Ppargc1b*, which encodes PGC-1β, a master regulator of mitochondrial metabolism and biogenesis, is significantly down-regulated. While we found that PGC-1β ectopic expression partially rescued osteoclastogenesis, PGC-1β is not a rate-limiting mediator of KDM5C-mitochondrial metabolism on its own. Notably, expression of the iron transporter TfR1 (*Tfrc*) was reduced in the absence of KDM5C; this suggests that the KDM5C-dependent iron uptake mechanism and PGC-1β expression may synergize to promote mitochondrial metabolism and osteoclastogenesis (*27*).

Our data showed that KDM5C-deficient male cells also have reduced osteoclastogenesis and expression of mitochondrial metabolism genes but not to the same extent as females. However, these differences did not translate into a difference in bone mass. These data suggest that there may be a gene dosage effect of KDM5C on gene expression and that KDM5D may play an important role in osteoclastogenesis and bone mass in males. KDM5D is also a lysine histone demethylase; however, it is less well-studied compared to KDM5C.The functional discrepancy between KDM5C and KDM5D paralogs was also found in other settings. For example, males with X-linked immunodeficiency caused by KDM5C variants have a more severe phenotype compared to heterozygote females, although each of them carries one functional copy of KDM5D and KDM5C, respectively. These observations suggest that KDM5C and KDM5D are not completely redundant (*33*). The unique and redundant roles of KDM5C/D and gene dose effects in bone mass regulation deserve further studies. We also did not see short stature on our KDM5C-deficient male mice, as seen in patients and cytomegalovirus-Cre-drive deletion of KDM5C (*18*), suggesting that the role of KDM5C in height is in cells of nonhematopoietic origin.

Osteoclastogenesis requires robust mitochondrial metabolism (*23*, *24*). We found that expression of mitochondrial genes increases during OC differentiation. PGC1-β promotes mitochondrial biogenesis and, coupled with iron transported by TfR1, increases mitochondrial respiration. PGC1-β and mitochondrial respiration also regulate actin cytoskeletal organization and therefore could also be involved in organization of glycolytic pathway enzymes (*23*, *29*). Further, it has been shown that estrogens decrease Oc.N by reducing OXPHOS in OC precursors (*25*). Glucose metabolism, both fermentation to lactate and oxidation in tricarboxylic acid (TCA) cycle, is also necessary for OC differentiation (*24*). While our study here was focused on ETC activity, it is possible that decreased flux of metabolites through the TCA cycle also contributes to decreased OXPHOS and ATP production. We did observe a decrease in glucose fermentation (measured by ECAR), suggesting that glucose metabolism is reduced. Future work examining the fates of major carbon sources such as glucose, glutamine, and amino acids will determine to what extent the metabolic defects we have observed are due to altered metabolic wiring versus transcriptional programming.

KDM5C gene dose has been associated with increased adiposity in females due to increased expression (*13*). Link *et al*. (*13*) found a strong correlation in humans between body mass index (BMI) and KMD5C expression and associations of noncoding variants with adiposity traits. It is not known whether KDM5C affects cellular metabolism in adipocytes. However, these findings, together with ours, suggest that KDM5C may have a broader role in metabolism beyond osteoclastogenesis.

Here, we find evidence that KDM5C is essential for monocyte differentiation to OCs. Overall, our findings highlight an epigenetic mechanism that controls osteoclastogenesis by governing the transcriptional programming of energy metabolism, positioning KDM5C as a potential target for the treatment of osteoporosis in females.

## MATERIALS AND METHODS

### Mice

The *Kdm5c*^fl/fl^ mouse with C57BL/6 background was a gift from Y. Shi’s laboratory. Briefly, exons 11 and 12 of *Kdm5c* were flanked by *flox* sequences to enable excision by Cre recombinase (*10*, *17*). *Kdm5c*^fl/fl^ mice were crossed to *Vav*-*iCre* (strain 008610) and *LysM*-*Cre* (strain 004781) mice, respectively, purchased from The Jackson Laboratory to generate KDM5C knockout mice. *Vav*-*iCre* is turned on at embryonic day 11.5 in almost all hematopoietic cells (*33*). Whereas *LysM*-*Cre* is turned on in myeloid progenitors at the granulocyte-monocyte progenitor stage (*34*). No reports have ever shown that express which of these two Cre would affect bone phenotypes (*35*–*37*). All mice were maintained following guidelines of the Institutional Animal Care and Use Committee at Van Andel Institute. Mice euthanization was conducted according to American Veterinary Medical Association Guidelines for the Euthanasia of Animals.

### Bone histomorphometry

Bone histomorphometry was performed as described before (*38*). Briefly, paraffin-embedded femurs from 2-month-old mice were sent to Pathology and Biorepository Core at Van Andel Research Institute for sectioning. TRAP staining on paraffin sections were performed using an acid phosphatase, leukocyte kit (Sigma-Aldrich, 387A) by following the standard protocol. N.Oc/BS, Oc.S/BS, and Oc.S/N.Oc were quantified using BioQuant osteo software (Nashville, TN, USA).

### Microcomputed tomography

Mouse femurs were scanned following the Journal of Bone and Mineral Research (JBMR)-recommended micro-CT guidelines as previously described (*39*). Reconstruction of cross-sectional slices was performed using NRecon software (SkyScan), while bone parameters were calculated through CTAN and CTVOL software. Bone parameters calculated are BV/TV, Tb.Th, Tb.Sp, Tb.N, cortical bone area (Ct.Ar), cortical thickness, the Ct.Ar/total cross-sectional area (Tt.Ar) ratio, and Tt.Ar.

### BMM isolation and culture

Bone marrow cells were collected from femurs and tibias and plated in complete BMM media [α-minimum essential medium plus 10% fetal bovine serum, 1% penicillin-streptomycin, 1% l-glutamine, and macrophage colony-stimulating factor (30 ng/ml)]. The next day, nonadherent cells were transferred to new dishes for BMM culture with fresh complete BMM media and expanded for 48 hours.

### Human PBMC extraction and culture

Human blood collection was approved by the Internal Review Board (IRB) of Calvin University (reference number: 20-023). All participating donors have understood and signed IRB-approved consent forms. PBMCs were obtained by centrifugation of blood through a Ficoll-Hypaque density gradient at 300*g* for 60 min. Adherent cells from PBMCs were cultured in BMM media for 1 to 3 days before use.

### Ex vivo osteoclastogenesis assay

BMM or cultured human monocytes (3 × 10^4^ or 6 × 10^4^ cells per well, respectively) were seeded into 96-well plates and cultured overnight with complete BMM media. Differentiation was induced by osteoclastogenic medium (50 ng/ml RANKL in complete BMM media), and induction medium was changed every other day. Cells were subjected to staining when OC fusion appeared (3 to 5 days for BMM and 7 to 9 days for human monocytes). TRAP staining was conducted to visualize OCs using an Acid Phosphatase Leukocyte (TRAP) Kit (Sigma-Aldrich) by following the standard protocol. Cells positive for TRAP staining and contain >3 nuclei were counted as OCs. Osteoclast numbers and area were quantified using ImageJ.

### Total RNA isolation and qRT-PCR

Total RNA from BMM were extracted using TRIzol reagent (Invitrogen) by following the standard protocol. Genomic DNAs were removed by using the DNA-free DNA Removal kit (Thermo Fisher Scientific, AM1906) according to the manufacturer’s instruction. Five hundred nanograns of RNA was subjected to the synthesis of first-strand cDNA using the SuperScript VILO cDNA synthesis Kit (Invitrogen, 11754050). Quantitative polymerase chain reaction (qPCR) was performed on a StepOne PCR instrument using SYBR Green qPCR Master Mix (Invitrogen, 4472908). Primers used for quantitative reverse transcription PCR (qRT-PCR) in this study are as follows: *Kdm5c*: 5′-GAGCAGTCTGTACTGTGCCA-3′ (forward), 5′-ATTCCCACATACAGCCACGG-3′ (reverse); *Nfatc1*: 5′-TCATCGGCGGGAAGAAGATG-3′ (forward), 5′-GTCCCGGTCAGTCTTTGCTT-3′ (reverse); *Fos*: 5′-TACTACCATTCCCCAGCCGA-3′ (forward), 5′-GCTGTCACCGTGGGGATAAA-3′ (reverse); *Oscar*: 5′-CGTGCTGACTTCACACCAAC-3′ (forward), 5′-GGTCACGTTGATCCCAGGAG-3′ (reverse); *Dcstamp:* GCTGTATCGGCTCATCTCC*T*-3′ (forward), 5′-ATGGACGACTCCTTGGGTTC-3′ (reverse); *Ocstamp:* 5′-AGCCACGGAACACCTCTTTG-3′ (forward), 5′-TGGAACAACTGCCTTGCAGA-3′ (reverse); *Ctsk*: 5′-GAAGGGAAGCAAGCACTGGA-3′ (forward), 5′-CCATGTTGGTAATGCCGCAG-3′ (reverse); *Acp5*: 5′-CTGGTATGTGCTGGCTGGAA-3′ (forward), 5′-CGCAAACGGTAGTAAGGGCT-3′ (reverse); *Atp6v0d2*: 5′-GGGCCTGGTTCGAGGATG-3′ (forward), 5′-GAAGTTGCCATAGTCCGTGGT-3′ (reverse); *Uqcrc2*: 5′-GCAACTGCTAGAGCCATGAAG-3′ (forward), 5′-TTAACCTTCGGGGCAACTTTGA-3′ (reverse); *Apt1b3*: 5′-TGCTGGAACCAGGAACCTAAA-3′ (forward), 5′-CTAGGCTCGTGCTGTGACTT-3′ (reverse); *Cox6b1*: 5′-CGCTACTCCGGGACAATCTT-3′ (forward), 5′-TCTGGTTCTGGTTGGGGAAG-3′ (reverse); *Sdhb*: 5′-CGTTCTCGGCAGAGTCGG-3′ (forward), 5′-GGTCCCATCGGTAAATGGCA-3′ (reverse); *Sdha*: 5′-TATGGTGCAGAAGCTCGGAAG-3′ (forward), 5′-ACTCATCGACCCGCACTTTG-3′ (reverse); *Ppargc1b*: 5′-CAGTACAGCCCCGATGACTC (forward), 5′-TTCGTAAGCGCAGCCAAGA-3′ (reverse); *Ppia*: 5′-AGCATACAGGTCCTGGCATC-3′ (forward), 5′- TTCACCTTCCCAAAGACCAC -3′ (reverse). *Ppia* gene was used as the internal control for normalization. The mRNA levels of genes of interest were normalized to the average levels of *Ppia*, and relative expression was calculated as the ratio to control (defined as 1).

### RNA-seq and data processing

BMM from fCtrl and f*Kdm5c*^ΔVav^ (*n* = 3) mice were seeded into 12-well plates followed by RANKL treatment for 0 hours (untreated), 16 hours, or 32 hours. Total RNA was extracted using the same method as described in qRT-PCR and sequenced by the Genomics Core at Van Andel Research Institute. Raw reads of RNA-seq data were mapped to *Mus musculus* (mm10). Subjunc v1.6.4 and featureCounts v1.6.4 were used to estimate the read counts on transcripts/gene exons. EdgeR v3.32.1 was used to identify differentially expressed genes. Genes with false discovery rate ≤ 0.05 (Benjamini-Hochberg–adjusted *P* values) were annotated as a differentially expressed gene between fCtrl and f*Kdm5c*^ΔVav^ BMM. R package enrichR v3.1 was used to identify gene sets (Gene Ontology Biology Process 2021) enriched in the differentially expressed genes. Terms with *P* < 0.05 were considered significant. The top five terms ranked by combined score of up- and down-regulated genes are listed in Fig. 4A. Combined score is computed using the logarithm of Fisher exact test *P* value multiplex by *z* score.

### Western blots

Cultured BMM were lysed in CHAPS buffer (Thermo Fisher Scientific) containing protein inhibitors. Ten to 40 μg of protein per sample was loaded to 10% SDS–polyacrylamide gel electrophoresis gels followed by transfer to polyvinylidene difluoride membranes. The membranes were then blocked with 5% nonfat milk in 1× tris-buffered saline plus 0.05% Tween 20 for 2 hours at room temperature and incubated with corresponding primary antibodies. Primary antibodies are as follows: anti-KDM5C (A301-034A, Bethyl Laboratories) or anti–β-actin (4967, Cell Signaling Technology) and OXPHOS rodent Western blots antibody cocktail (45-8099, Thermo Fisher Scientific). Blots were developed using the SuperSignalt West Dura Extended Duration Substrate (Thermo Fisher Scientific) and imaged using the ChemiDoc MP Imaging System (Bio-Rad).

### Extracellular flux assay

Seahorse assay and ATP calculations were performed as described (*37*, *40*, *41*). OCR and ECAR of cultured BMM were measured using the Seahorse XF96 Extracellular Flux Analyzer. Briefly, 20,000 BMM were seeded to XF96 plates and treated with RANKL to induce osteoclastogenesis. Three days after RANKL treatment, cellular bioenergetics were assessed using the Seahorse XF Cell Mito Stress Test Kit with sequential addition of 1.5 μM oligomycin (inhibiting ATP synthesis), 3 μM carbonyl cyanide 4-(trifluoromethoxy) phenylhydrazone (uncoupling), 0.5 μM rotenone/antimycin A (inhibiting respiratory chain complexes I and III, respectively), and 10 mM monensin (stimulating Na + pumps on plasma membrane). Data were normalized to number of cells.

### Mitochondrial ETC inhibition

Mitochondrial ETC complex I inhibitor piericidin A (#15379) and complex II inhibitor atpenin A5 (#11898) were purchased from Cayman Chemical, while complex III inhibitor myxothiazol (T5580) was purchased from MilliporeSigma. Cultured BMM were treated with DMSO (control group), piericidin A (0.2 μM), atpenin A5 (0.4 μM), myxothiazol (0.2 μM), or antimycin (0.2 μM) along with methyl-pyruvate (1 mM) and uridine (400 μM) followed by RANKL stimulation. All concentrations were referred to published papers (*42*, *43*) and optimized for BMM treatment. TRAP staining was performed when mature OCs appeared in the control group.

### BMM retroviral transfection

BMM were transduced with control (pMSCV-ires-Thy1.1; pMIT) or PGC-1β–expressing (pMiT- *Pgc1-*β) retrovirus, as previously described (*44*, *45*). Briefly, 293T cells were transfected with vectors and Lipofectamine 2000 to generate retroviral supernatants. BMM were cultured in six-well plates with complete BMM media until 70% confluency was reached. Cells were then transfected with pMiT or pMiT-*Pgc1-*β retrovirus containing supernatants by centrifuging under 2500 rpm for 60 min at 30°C before being cultured in fresh complete BMM media. Two days after transfection, cells were digested with 0.5 mM EDTA, stained with phycoerythrin (PE)–Thy-1.1 antibody (12-0900-81, Thermo Fisher Scientific), and sorted using anti-PE microbeads (Miltenyi Biotec). Sorted BMM were seeded in 96-well plates for osteoclastogenesis, in 12-well plates for RNA extraction and Western blots, or in XF96 plates for Seahorse assays.

### KDM5 inhibitor treatment

The KDM5i, KDM5A-IN-1, was purchased from MedChemExpress (catalog no. HY-100014). The half maximal effective concentration was defined as concentration at which the osteoclast numbers and area are halved compared to the control group. Cultured BMM from fCtrl mice or human monocytes were thereafter treated with DMSO or KDM5i for osteoclastogenesis assay, Seahorse assay, or RNA extraction.

### Statistical analysis

All data were analyzed using GraphPad Prism software (version 9). An unpaired or paired Student’s *t* test was performed for experiments with two groups for statistical significance calculation. A one-way or two-way analysis of variance (ANOVA) was performed to determine statistical significance between multiple groups. Actual *P* values are shown on each graph.
