# Supplementary material for: The histone demethylase KDM5C controls female bone mass by promoting energy metabolism in osteoclasts
Source: Sci Adv. 2023 Apr 5;9(14):eadg0731. doi: 10.1126/sciadv.adg0731 (PMC10075994; doi:10.1126/sciadv.adg0731)
Supplement: Supplementary file 1 — Figs. S1 to S8 [file sciadv.adg0731_sm.pdf]

Science Advances
AAAS

Supplementary Materials for

The histone demethylase KDM5C controls female bone mass by promoting energy metabolism in osteoclasts

Huadie Liu *et al.*

Corresponding author: Tao Yang, tao.yang@vai.org; Connie M. Krawczyk, connie.krawczyk@vai.org

Sci. Adv. **9**, eadg0731 (2023)
DOI: 10.1126/sciadv.adg0731

This PDF file includes:

Figs. S1 to S8

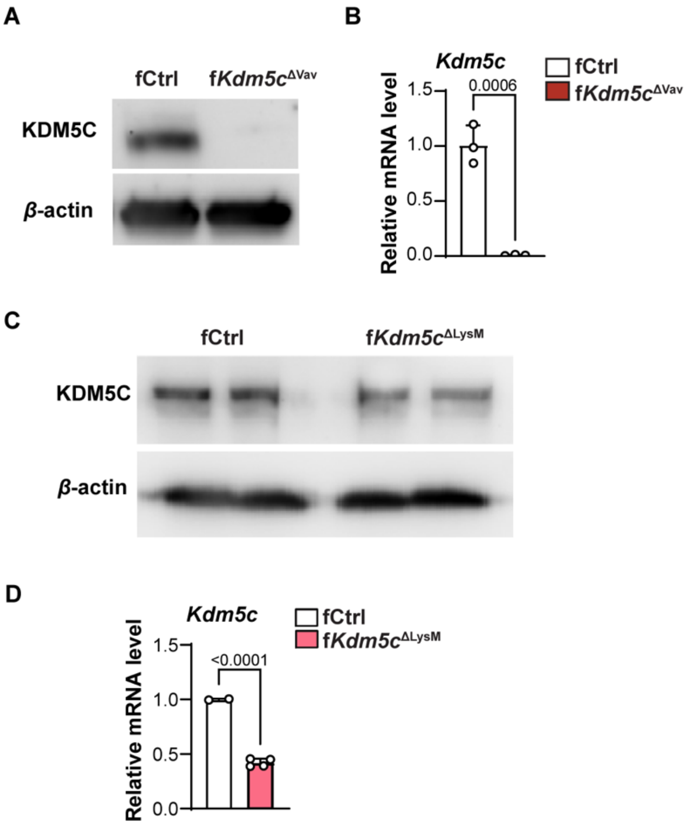

Fig. S1. Reduced deletion efficiency by *LysM-Cre* compared to *Vav-iCre* in BMM.

(**A**-**B**) Protein levels (A) and mRNA levels (B) of KDM5C in f*Kdm5c*$^{\Delta Vav}$ and fCtrl BMM. (**C**-**D**) Protein levels (C) and mRNA levels (D) of KDM5C in f*Kdm5c*$^{\Delta LysM}$ and fCtrl BMM. All data comparisons are conducted by Student's *t*-test, two-tailed. Data are presented as mean ± s.e.m.

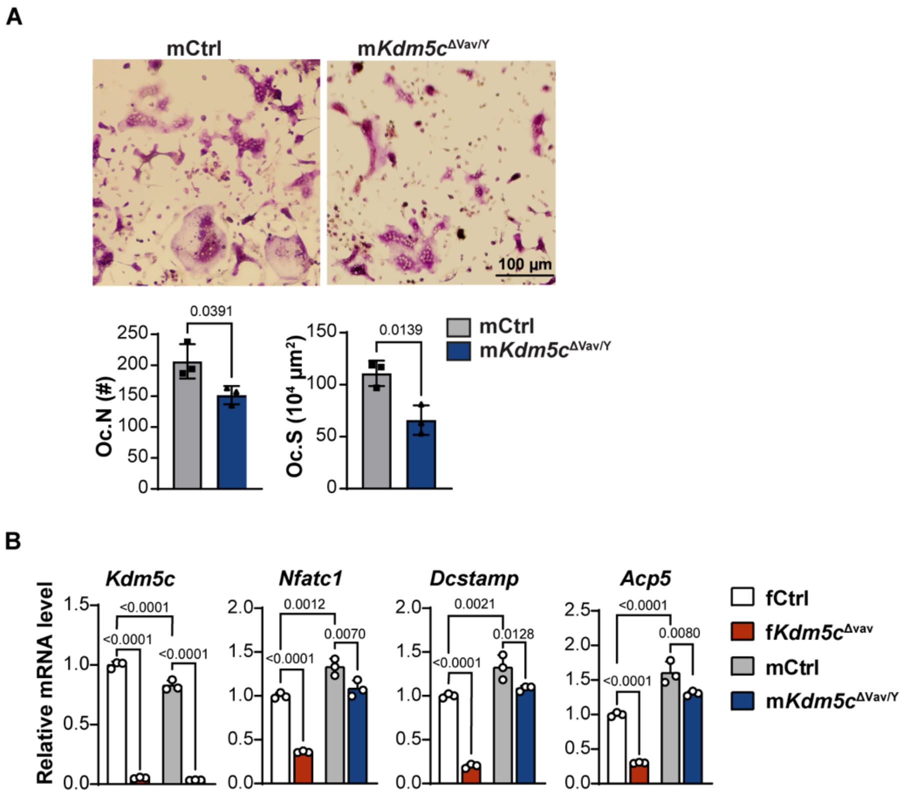

Fig. S2. Decreased osteoclastogenesis in male *Kdm5c*$^{\Delta\text{Vav/Y}}$ mice.

(**A**) *Ex vivo* osteoclastogenesis on BMM of mCtrl (m*Kdm5c*$^{\text{wt/Y}}$ Cre+) and m*Kdm5c*$^{\Delta\text{Vav/Y}}$ mice. Oc.N and Oc.S were measured and calculated (n=3 per genotype). Scale bar, 100 µm. (**B**) mRNA levels of osteoclastogenic genes were detected by qRT-PCR in fCtrl, f*Kdm5c*$^{\Delta\text{Vav}}$, mCtrl, and m*Kdm5c*$^{\Delta\text{Vav/Y}}$ BMM 48 hours after RANKL stimulation. All comparisons are conducted by Student's *t*-test, two-tailed. Data are presented as mean ± s.e.m.

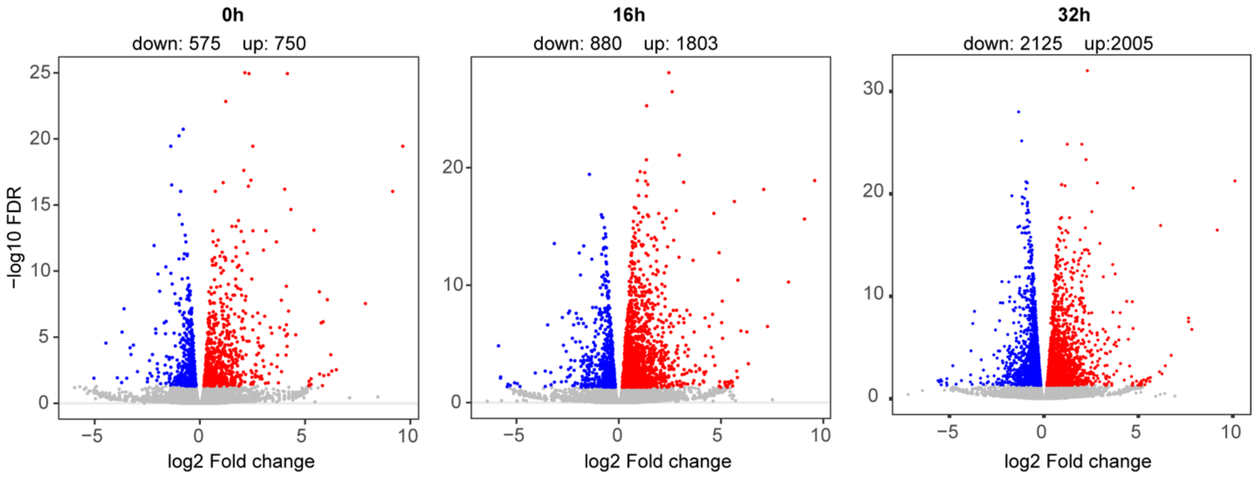

Fig. S3. Transcriptomic changes between f*Kdm5c*$^{\Delta Vav}$ and fCtrl BMM during *ex vivo* osteoclastogenesis assay. Volcano plots of RNA-seq data showing significant changes in gene expression between f*Kdm5c*$^{\Delta Vav}$ and control BMM at different osteoclastogenesis stages (0h, 16h, and 32h after RANKL stimulation).

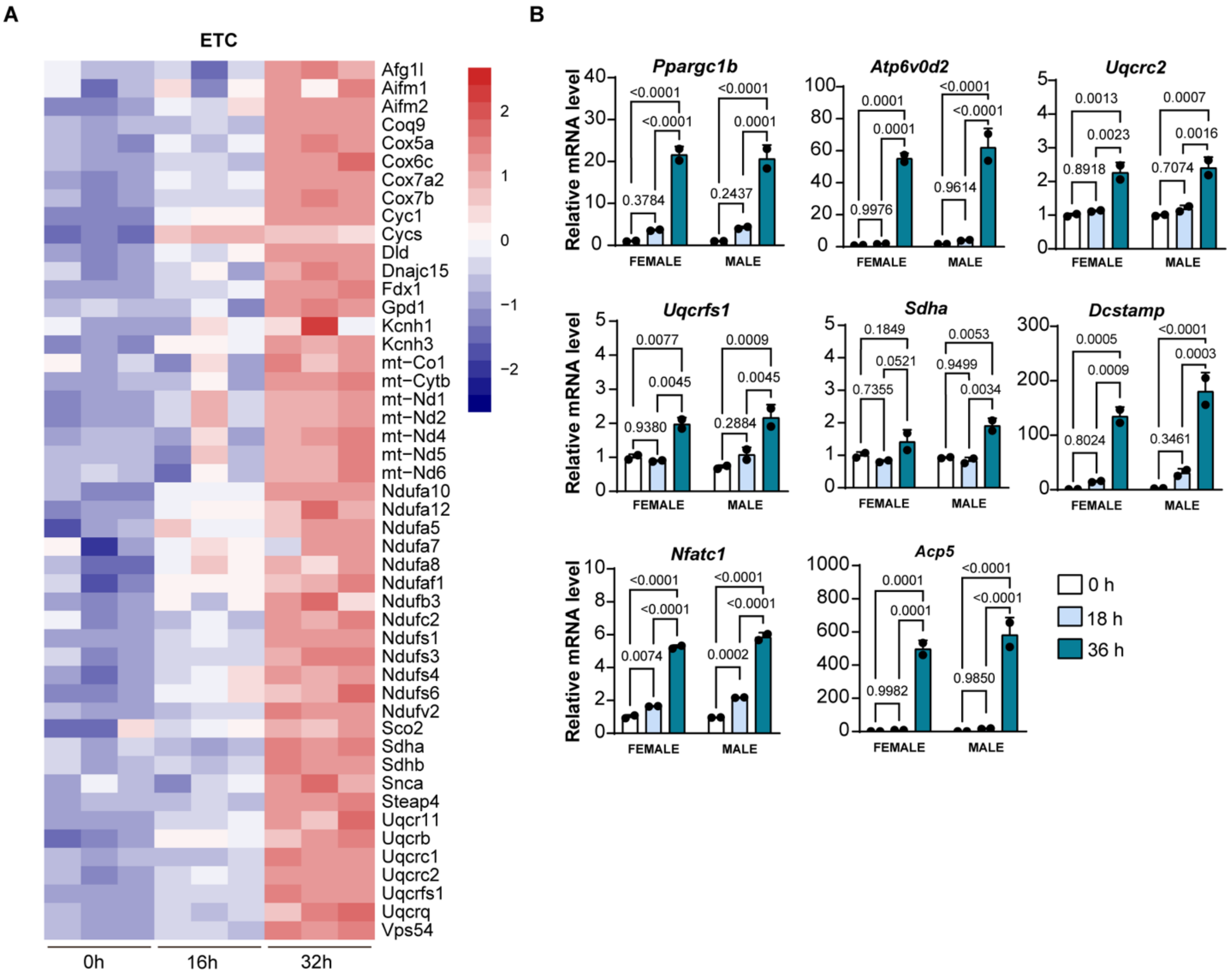

Fig. S4. Upregulation of mitochondrial genes during osteoclastogenesis.

(**A**) Heatmap of mitochondrial ETC genes expression in fCtrl BMM at different osteoclastogenesis stages (0h, 16h, and 32h of RANKL stimulation). Results are analyzed from RNA sequencing data. (**B**) mRNA levels of mitochondrial ETC and osteoclasteogenic genes detected by qRT-PCR in both fCtrl and mCtrl BMM at osteoclastogensis stages (0h, 18h, and 36h of RANKL stimulation). Statistics in (B) are conducted by two-way ANOVA plus šídák's multiple comparisons test. Data are presented as mean ± s.e.m.

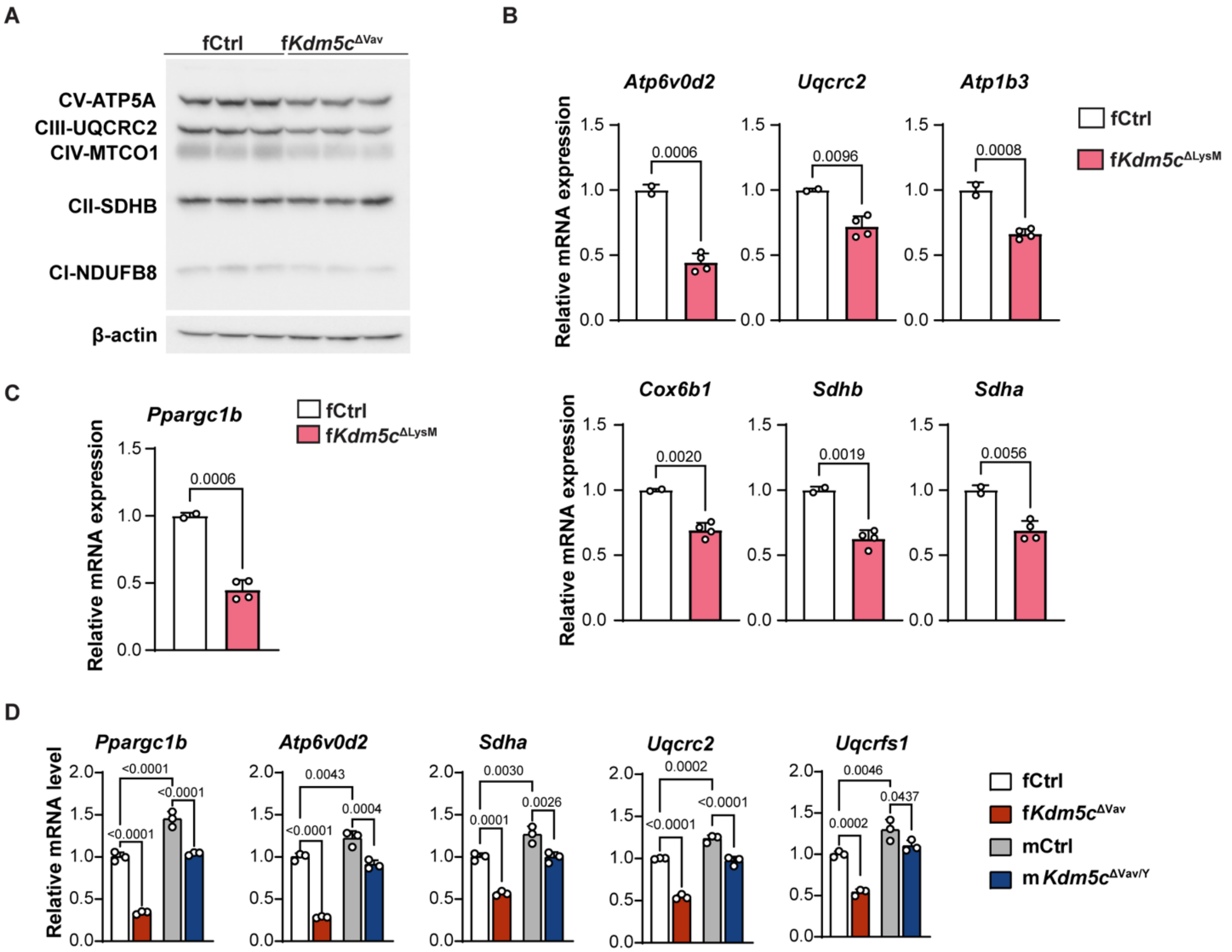

Fig. S5. ***Ppargc1b* and mitochondrial respiration genes are repressed in KDM5C-deficient BMM.**

(**A**) Lower exposure of Fig.4E of mitochondrial OXPHOS complex proteins in f*Kdm5c*$^{\Delta Vav}$ and fCtrl BMM (**B**) mRNA levels of mitochondrial respiration genes in f*Kdm5c*$^{\Delta LysM}$ and fCtrl BMM. (**C**) Level of *Ppargc1b* mRNA in f*Kdm5c*$^{\Delta LysM}$ and fCtrl BMM. (**D**) Comparison of mRNA levels of mitochondrial genes in fCtrl, f*Kdm5c*$^{\Delta Vav}$, mCtrl and m*Kdm5c*$^{\Delta Vav/Y}$. Data comparisons in (B and C) are conducted by Student's *t*-test, two-tailed. Statistics in (D) are by Ordinary one-way ANOVA. Data are presented as mean ± s.e.m.

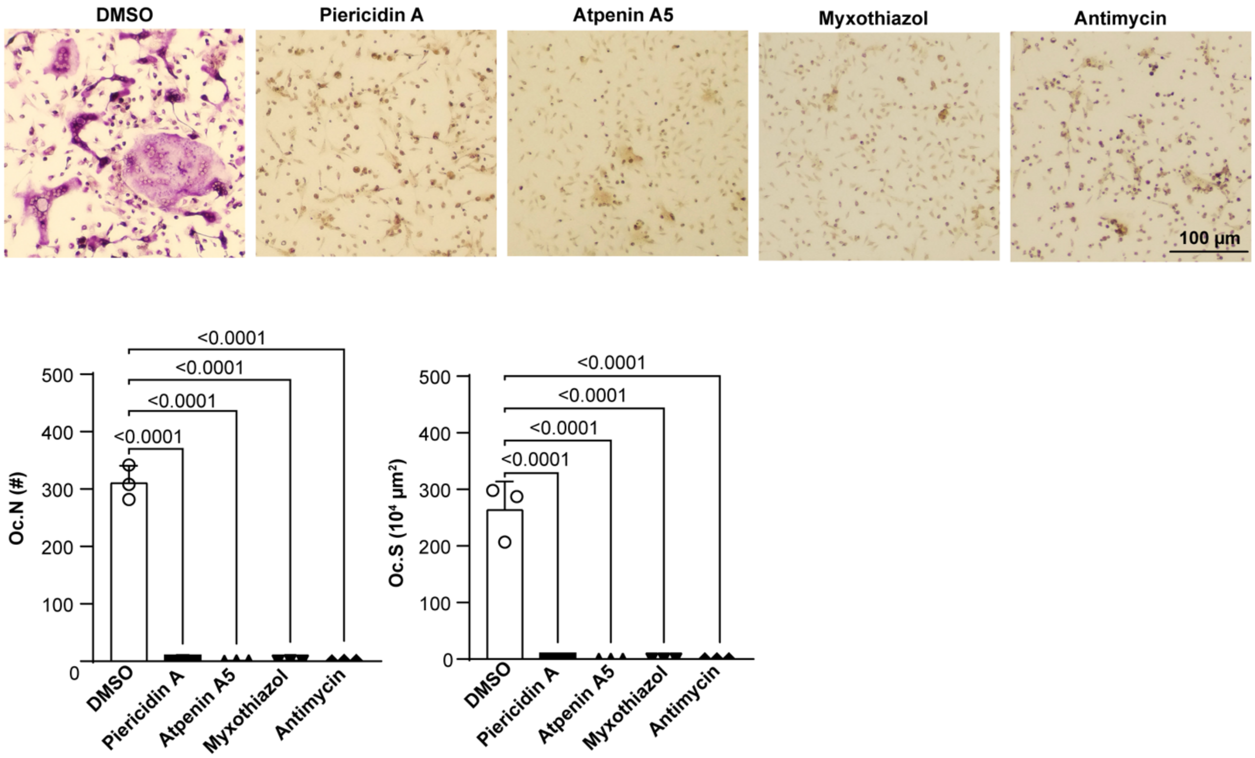

Fig. S6. Inhibition of mitochondrial ETC complexes completely blocks osteoclast formation.

TRAP staining of fCtrl BMM treated with DMSO (control group), Piericidin A (complex l inhibitor), Atpenin A5 (complex ll inhibitor), Myxothiazol (complex lll inhibitor), and antimycin (complex lll inhibitor). Oc.N and Oc.S were measured and calculated (*n*=3 per genotype). All data comparisons are conducted by one-way ANOVA analysis. Scale bar, 100 µm. Data are presented as mean ± s.e.m.

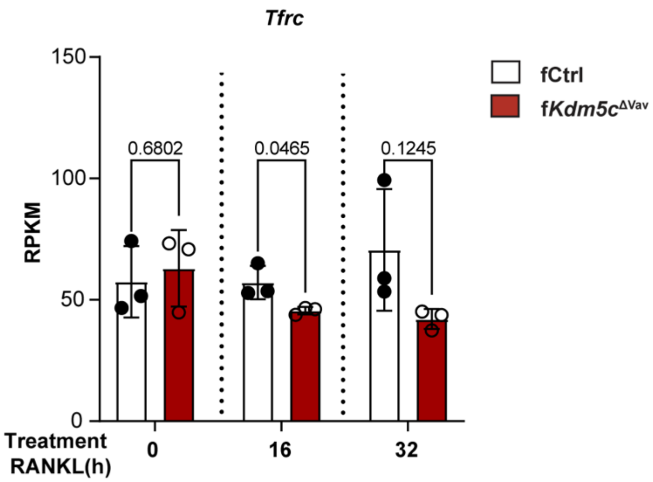

Fig. S7. KDM5C-deficeint BMM have decreased transferrin receptor gene expression during osteoclastogenesis.

Level of Transferrin receptor protein 1 gene (*Tfrc1*) transcripts in fCtrl and f*Kdm5c*$^{\Delta Vav}$ BMM during osteoclastogenesis (Student's *t*-test, two-tailed). Data are presented as mean ± s.e.m.

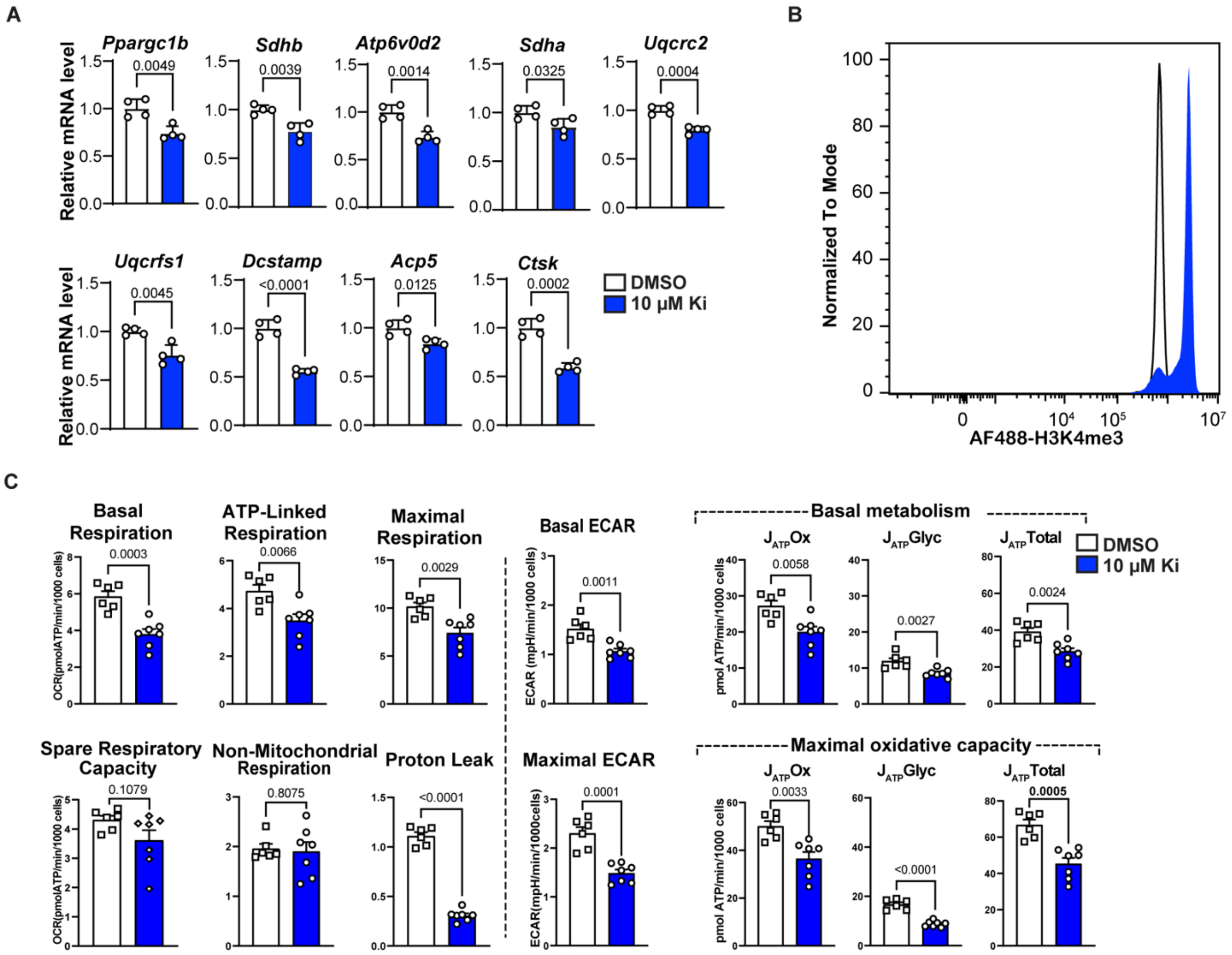

Fig. S8. Pharmacological inhibition of KDM5 increases H3K4me3 and impairs mitochondrial metabolism and osteoclastogenesis of mouse and human monocytes.

(**A**) Expression of *Ppargc1b,* osteoclast and mitochondrial respiration genes in DMSO and Ki treated mouse BMM. mRNA was detected by qRT-PCR 48h after RANKL treatment. (**B**) H3K4me3 levels in control and Ki treated CD14+ monocytes cultured from human peripheral blood. H3K4me3 levels were detected by flow cytometry 24h after inhibitor treatment. (**C**) Detailed parameters of OCR, ECAR and Oxidative/glycolytic/total ATP production in control and Ki-treated human PBMC-monocyte 5 days after osteogenic induction. Comparisons in (A and C) are conducted by Student's *t*-test, two-tailed. Data are presented as mean ± s.e.m.
